# Development and validation of multiplex real-time PCR for simultaneous detection of six bacterial pathogens causing lower respiratory tract infections and antimicrobial resistance genes

**DOI:** 10.1186/s12879-024-09028-2

**Published:** 2024-02-07

**Authors:** Tran Thi Ngoc Dung, Voong Vinh Phat, Chau Vinh, Nguyen Phu Huong Lan, Nguyen Luong Nha Phuong, Le Thi Quynh Ngan, Guy Thwaites, Louise Thwaites, Maia Rabaa, Anh T. K. Nguyen, Pham Thanh Duy

**Affiliations:** 1 Molecular Epidemiology Group, Oxford University Clinical Research Unit, 764 Vo Van Kiet Street, Ward 1, District 5, Ho Chi Minh City, Vietnam; 2 Hospital for Tropical Diseases, Ho Chi Minh City, Vietnam; 3 Centre for Tropical Medicine and Global Health, Nuffield Department of Clinical Medicine, Oxford University, Oxford, UK

**Keywords:** Real-time PCR, Lower respiratory tract infections, Antimicrobial resistance, Melting curve analysis, Molecular diagnostics

## Abstract

**Background:**

*Klebsiella pneumoniae, Acinetobacter baumannii, Pseudomonas aeruginosa*, *Escherichia coli, Streptococcus pneumoniae and Staphylococcus aureus* are major bacterial causes of lower respiratory tract infections (LRTIs) globally, leading to substantial morbidity and mortality. The rapid increase of antimicrobial resistance (AMR) in these pathogens poses significant challenges for their effective antibiotic therapy. In low-resourced settings, patients with LRTIs are prescribed antibiotics empirically while awaiting several days for culture results. Rapid pathogen and AMR gene detection could prompt optimal antibiotic use and improve outcomes.

**Methods:**

Here, we developed multiplex quantitative real-time PCR using EvaGreen dye and melting curve analysis to rapidly identify six major pathogens and fourteen AMR genes directly from respiratory samples. The reproducibility, linearity, limit of detection (LOD) of real-time PCR assays for pathogen detection were evaluated using DNA control mixes and spiked tracheal aspirate. The performance of RT-PCR assays was subsequently compared with the gold standard, conventional culture on 50 tracheal aspirate and sputum specimens of ICU patients.

**Results:**

The sensitivity of RT-PCR assays was 100% for *K. pneumoniae*, *A. baumannii*, *P. aeruginosa*, *E. coli* and 63.6% for *S. aureus* and the specificity ranged from 87.5% to 97.6%. The kappa correlation values of all pathogens between the two methods varied from 0.63 to 0.95. The limit of detection of target bacteria was 1600 CFU/ml. The quantitative results from the PCR assays demonstrated 100% concordance with quantitative culture of tracheal aspirates. Compared to culture, PCR assays exhibited higher sensitivity in detecting mixed infections and *S. pneumoniae*. There was a high level of concordance between the detection of AMR gene and AMR phenotype in single infections.

**Conclusions:**

Our multiplex quantitative RT-PCR assays are fast and simple, but sensitive and specific in detecting six bacterial pathogens of LRTIs and their antimicrobial resistance genes and should be further evaluated for clinical utility.

**Supplementary Information:**

The online version contains supplementary material available at 10.1186/s12879-024-09028-2.

## Background

Lower respiratory tract infections (LRTIs) are a leading infectious cause of morbidity and mortality globally. It is estimated that 336 million episodes of LRTIs occurred in 2016, resulting in nearly 2.4 million deaths of all ages [1]. Although viruses such as influenza viruses and respiratory syncytial viruses are responsible for a large proportion of LRTIs, most deaths are caused by bacterial agents, including *Streptococcus pneumoniae, Haemophilus influenzae, Staphylococcus aureus, Pseudomonas aeruginosa, Klebsiella pneumoniae, Escherichia coli* and *Acinetobacter baumannii* [2–4]. In recent years, the worldwide increase of antimicrobial resistance (AMR) in respiratory bacterial pathogens threatens the effectiveness of antibiotic treatment [5]. Multidrug-resistant (MDR) Gram-negative pathogens are more frequently identified in patients with LRTIs, especially in ICUs, leading to increased risks of poor outcomes, prolonged hospital stay, and mortality [6–8]. Early and rapid detection of LRTI pathogens and their AMR phenotype is key to informing appropriate antibiotic therapy and reducing risks of severe complications and mortality [3, 9, 10].

In recent years, the worldwide increase of antimicrobial resistance (AMR) in respiratory bacterial pathogens threatens the effectiveness of antibiotic treatment [5]. Multidrug-resistant (MDR) Gram-negative pathogens are more frequently identified in patients with LRTIs, especially in ICUs, leading to increased risks of poor outcomes, prolonged hospital stays, and mortality [6–8]. Early and rapid detection of LRTI (Lower Respiratory Infection) pathogens and their AMR phenotype are key to inform appropriate antibiotic therapy and reduce risks of severe complications and mortality [3, 9, 10]. Currently, a variety of traditional diagnostic methods are used to detect respiratory pathogens, including microscopic examination, bacterial culture and antigen detection [11]. These methods often had low sensitivity and long turnaround time [11]. Recently, several molecular PCR-based assays have been developed for the diagnostics of respiratory pathogens. Luminex assays and TaqMan array cards can detect many viruses and bacteria simultaneously [12, 13]. The BioFire FilmArray Pneumonia Plus Panel can detect 18 bacteria (11 Gram-negative, 4 Gram-positive and 3 atypical)**,** 7 AMR markers, and 9 viruses that cause pneumonia [14]. However, the FilmArray assay is expensive and may not be affordable for routine diagnostics, particularly in low- and middle-income (LMIC) settings [15]. Currently, a variety of traditional diagnostic methods are used to detect respiratory pathogens, including microscopic examination, bacterial culture and antigen detection [11]. These methods often had low sensitivity and long turnaround times [11]. Recently, several molecular PCR-based assays have been developed for the diagnostics of respiratory pathogens. Luminex assays and TaqMan array cards can detect many viruses and bacteria simultaneously [12, 13]. The BioFire FilmArray Pneumonia Plus Panel can detect 18 bacteria (11 Gram-negative, 4 Gram-positive and 3 atypical)**,** 7 AMR markers, and 9 viruses that cause pneumonia [14]. However, the FilmArray assay is expensive and may not be affordable for routine diagnostics, particularly in low- and middle-income (LMIC) settings [15].

Alternatively, multiplex real-time PCR with either a fluorescent probe or fluorescent dye targeting key locally relevant bacterial-AMR gene combinations can provide a pragmatic and inexpensive diagnostic method for clinical use. Compared to probe-based assay, dye-based assay with melting curve analysis (MCA) is less expensive and can detect more targets. The most commonly used fluorescent dye for multiplex real-time PCR is SYBR Green. However, EvaGreen (EG), a third-generation, new saturating fluorescent dye are proved to be better than SYBR Green due to EG dye can be used at higher concentrations without inhibiting PCR and shows equal binding affinity for GC-rich and AT-rich regions [16]. EG-based multiplex real-time PCR with MCA has been developed for the detection of multiple respiratory pathogens [17, 18].

Here, we aimed to develop and validate EG-based multiplex quantitative real-time PCR with MCA (EG-mPCR assays) to detect six bacterial pathogens and fourteen AMR genes directly from respiratory specimens.

## Methods

### Bacteria strains

To develop and validate our EG-mPCR assays, ATCC strains, clinical strains and off-target controls (*Acinetobacter lwoffii, Staphylococcus epidermidis, Shigella sonnei, Salmonella typhi, Campylobacter jejuni*) were utilized. Standard strains of *E. coli* (ATCC25922), *K. pneumoniae* (ATCC700603), *S. pneumoniae* (ATCC49619), *S. aureus* (ATCC25923), *P. aeruginosa* (ATCC27853) were purchased from the American Type Culture Collection (ATCC, Manassas, VA, USA) and subsequently cultured in Luria-Bertani (LB) agar or nutrient agar (NA). Further, eight clinical strains carrying the target AMR genes were used as positive controls for AMR gene detection assays.

### Clinical sample preparation

The tracheal aspirates (TA) or sputum specimens collected from ICU patients, who were clinically diagnosed with LRTIs at the Hospital of Tropical Diseases in Ho Chi Minh City, were sent to Microbiology Department for culture. Residual samples from these respiratory specimens were transferred to the Molecular Laboratory at Oxford University Clinical Research Unit (OUCRU) for the evaluation of the EG-mPCR assays.

### Microbiological culture

The TA and the sputum samples were collected into a sterile container, followed by Gram staining and examination under direct microscopy. Samples having < 10 epithelial cells and > 25 leukocytes in each area upon 100 × magnification were considered good quality for bacterial culture and were included in this study. TA samples were liquefied with Sputasol liquid (Oxoid, USA) with a ratio of 1:1 and diluted with MRD (maximum recovery diluent) broth with ratio of 1:9 before microbiological testing.

Subsequently, 1 μl of suspension inoculated into blood agar, Macconkey agar and chocolate agar media (Oxoid, USA), and incubated for 24—48 h at 35–37 °C or at 35–37 °C with ~ 5% CO_2_. Bacterial identification and antimicrobial susceptibility testing (AST) were performed using MALDI-TOF (Bruker, Germany) and Vitek2 automatic identification and AST system (Biomérieux, France). Antimicrobial susceptibility results were interpreted according to the Clinical and Laboratory Standards Institute (CLSI) 2021 guidelines [19]. In TA culture, samples with bacterial growth ≥ 10^5^ CFU/mL were considered positive following the local guidelines for microbiological diagnostics.

### Extraction of nucleic acids

DNA extraction from bacterial isolates was performed using the Wizard Genomic DNA Extraction Kit (Promega, Fitchburg, USA) according to the manufacturer’s instructions. The quality and concentration of the DNA were assessed using a Nano-drop spectrophotometer prior to PCR amplification.

For TA/sputum samples, an aliquot of 200 μl was homogenized with 4 times the volume of 0.1% dithiothreitol (DTT) for 15 min at room temperature and centrifuged at 8000 rpm for 10 min. Subsequently, the supernatant was discarded, and the pellet was resuspended with 200 μl PBS. Next, 25 μl of 10X buffer (200 mM Tris–HCl, 0.2 μl of Benzonase (Sigma) and 24.8 μl of sterilized water) were added, followed by 2-h incubation at 37 °C. The mixture was centrifuged at 8000 rpm for 10 min and the pellet was subsequently resuspended in a mixture of 4 μl EDTA, 120 μl NaCl and 76 μl H_2_O. Centrifugation was performed again at 8000 rpm for 10 min and the pellet was resuspended in 200 μl of TE before DNA extraction. 200 μl of pre-treated samples were subjected to an automated extraction on a MagNA Pure 96 nucleic extraction system (Roche Applied Sciences, UK), according to the manufacturer's recommendations. For each DNA extraction batch, we incorporated one culture-positive and one culture-negative tracheal aspirate sample as positive and negative controls.

### Primer design

Twenty primer sets were designed based on the conserved regions which can produce amplicons having melting temperatures (*T*_*m*_s) ranging from 75 °C to 92 °C and target-specific *T*_*m*_ values differed from each other by at least 1 °C. The primer sequences are described in Table 1. The actual *T*_*m*_ of all primers was determined following the performance of singleplex PCRs and multiplex PCRs.

**Table 1:** Sequences of primers used for singleplex and multiplex real-time EG-PCR assays

Primer	Gene	Sequence (5'-3')	Final conc.(μM)	Amplicon size (bp)	Reference
**Pathogen detection assays**					
A. baumannii-F	ompA	GTTAAAGGCGACGTAGACG	1	194	this study
A. baumannii-R		TCAGAAGTACCACGTGTACCA			
P. aeruginosa-F	oatA	GAACTGCTCTTCCACCGACA	0.6	170	this study
P. aeruginosa-R		GCTGGTTCCTGATGGAGCC			
K. pneumoniae-F	khe	AGGGTTCGAGTTTGCTGCT	1	171	this study
K. pneumoniae-R		AGACAACCGCGTAGGCATT			
E. coli-F	yaiO	TGATTTCCGTGCGTCTGAATG	1	115	[20]
E. coli-R		ATGCTGCCGTAGCGTGTTTC			
S. aureus-F	sa442	TCGGTACACGATATTCTTCAC	1.2	179	[18]
S. aureus-R		ACTCTCGTATGACCAGCTTC			
S. pneumoniae-F	ply	GAATTCCCTGTCTTTTCAAAGTC	0.6	106	this study
S. pneumoniae-R		GACCTTGTTAGAGAATAATCCCA			
**AMR gene detection assays**					
mecA-F	mecA	GGCATCGTTCCAAAGAATGT	0.8	128	this study
mecA-R		AGTGGAACGAAGGTATCATCTT			
ermB-F	ermB	GTCTCGATTCAGCAATTG	1.2	171	this study
ermB-R		CAGTTGACGATATTCTCGAT			
OXA-48-F	bla_OXA-48_	GCGTGTATTAGCCTTATCGG	0.5	204	this study
OXA-48-R		GCGGGTAAAAATGCTTGGT			
CTX-M-9-F	bla_CTX-M-9_	GTGCTTTATCGCGGTGA	0.8	128	this study
CTX-M-9-R		GCAGGCTTGATCTCGACAGG			
SHV-F	bla_SHV_	GGTGGATGCCGGTGACGA	0.5	96	this study
SHV-R		TCGGCAAGGTGTTTTTCGC			
OXA-23-F	bla_OXA-23_	ACTTGCTATGTGGTTGCTTC	0.8	106	this study
OXA-23-R		GAATCACCTGATTATGTCCTT			
mcr-1-F	mcr-1	TCGTATCGCTATGTGCTAAAGC	0.5	128	this study
mcr-1-R		TCGGTCTGTAGGGCATTTTG			
TEM-F	bla_TEM_	CCCTTTTTTGCGGCATTTTGC	0.5	163	this study
TEM-R		TTGGAAAACGTTCTTCGGGG			
NDM-F	bla_NDM_	GACAATATCACCGTTGGGAT	0.5	113	this study
NDM-R		TAGTGCTCAGTGTCGGCAT			
mphA-F	mphA	AACTGTACGCACTTGCAG	0.5	179	this study
mphA-R		CAGCACCCGCGCCTCTGGTT			
IMP-F	bla_IMP_	GGTTTAAYAAAACAACCACC	0.5	189	this study
IMP-R		GGAATAGAGTGGCTTAAYTCTC			
VanA-F	VanA	TGTTTGGGGGTTGCTCAGA	0.5	190	this study
VanA-R		TATCCGGCGAGAGTACTGC			
CTX-M-1-F	bla_CTX-M-1_	ACCACCAACGATATCGCGG	0.5	143	this study
CTX-M-1-R		TACAAACCGTCGGTGACGAT			
KPC-F	bla_KPC_	TCGTCGCGGAACCATTC	0.8	121	this study
KPC-R		ACAGTGGGAAGCGCTCCTC			

For simultaneous detection of the six LRTI pathogens, six primer pairs including two previously published ones, were designed to target the known species-specific genes. These genes included *yaiO* for *E. coli* [20], *ompA* for *A. baumannii* [21], *oatA* for *P. aeruginosa* [22], *sa442* for *S. aureus* [18], *ply* for *S. pneumoniae* [18] and *khe* for *K. pneumoniae* [22]. Additionally, fourteen primers were also designed to identify fourteen common acquired AMR genes present in these bacteria, encoding resistance to β-lactams (*bla*_SHV_, *bla*_TEM_, *bla*_CTX-M-9,_ *bla*_CTX-M-1_), methicillin (*mecA*), carbapenems (*bla*_KPC_, *bla*_NDM_, *bla*_OXA-48_, *bla*_OXA-23,_ *bla*_*IMP*_), colistin (*mcr-1*), macrolides (*ermB, mphA*) and vancomycin (*vanA*). All primer pairs underwent a comprehensive search against the ARG-ANNOT (Antibiotic Resistance Gene-ANNOTation) database to ascertain their inclusivity across all variants.

### Development of EG-mPCR assays

Singleplex real-time PCR assays were performed in 20 μl reaction volume, which included 5 μl DNA template, 0.8 μl forward and reverse primers and 10 μl SensiFAST HRM kit (Meridian Bioscience), to check the actual *T*_*m*_s of each individual target amplicon. Sterile - purified water was used as the negative control. PCR amplifications were run on a LightCycler 480II (Roche applied sciences, UK) with the following thermal conditions: initial denaturation at 95 °C for 5 min, followed by 40 cycles of denaturation, 95 °C, for 10 s; annealing, 60 °C for 30 s; extension, 72 °C for 30 s. For multiplex real-time PCR assays, the component and thermal condition were the same as singleplex real-time PCRs, except the concentration of each primer pair varied from 0.25 μM to 0.5 μM. After PCR amplification, the melting curve analysis (MCA) was conducted in the same thermocycler at 65 °C to 95 °C; and cooling cycle at 37 °C for 30 s. Fluorescence was continuously measured and the melting temperature (*T*_*m*_) was calculated by plotting the negative derivative of fluorescence over temperature versus temperature (− d*F*/d*T* versus *T*). Conventional PCRs with a different set of primers were later performed to confirm the results of EG-mPCR assays.

The reproducibility, linearity, and limit of detection (LOD) of EG-mPCR assays for pathogen detection were evaluated using fivefold serial dilutions of two DNA control mixes. Each DNA control mix was prepared by pooling an equal volume of 10^8^ CFU/mL of each bacterium, followed by DNA extraction. Subsequently, fivefold serially diluted DNA standards, corresponding to the bacterial concentrations from 10^6^ to 12.8 CFU/mL, were prepared for the assays. The standard curves were performed on each EG-mPCR run. A reliable quantitative EG-mPCR reading was established when the linear coefficient *R*^2^ surpassed 0.8, signifying a robust linear relationship between the fluorescence intensity and the logarithm (log10) of the bacterial concentration. The efficiency of the two quantitative EG-mPCR assays was assessed by analyzing the Cp values derived from fivefold serial dilutions of DNA control mixes. The efficiency was calculated using the formula: Efficiency = 10^(−1/slope)^-1, following the methods of Rasmussen [23]. Assessment of non-specific amplification or interference was performed by comparing the PCR results obtained from singleplex versus multiplex assays.

To validate the assays in the TA specimen, 200 μl pre-treated culture-negative TA specimen was spiked with fivefold serial dilutions of bacteria (from 10^6^ to 12.8 CFU/mL), followed by DNA extraction using Roche’s MagNA Pure 96 system. The EG-mPCR assays were performed as described above.

### Diagnostic performance of EG-mPCR assays in comparison to conventional culture

Fifty respiratory samples, including 32 tracheal aspirates and 18 sputum specimens, were diagnosed using both the EG-mPCR assays and conventional culture. Singleplex EG-PCR and conventional PCR were employed to validate discrepancies between the culture and PCR results. The conventional multiplex PCR assays utilized the identical set of target genes as the EG-mPCR assays, albeit with distinct primer sequences (Table S1). The conventional PCR conditions involved an initial denaturation at 95 °C for 15 min, followed by 35 cycles consisting of a) denaturation at 95 °C for 30 s, b) primer annealing at 55 °C for 45 s, c) primer extension at 72 °C for 2 min, and a final extension at 72 °C for 10 min. In addition, the correlation between the presence of the AMR gene and AMR phenotype was also examined. The degree of agreement between EG-mPCR assays and conventional culture for bacterial identification was measured by Cohen’s kappa [24].

### Turnaround time

The turnaround time from sample collection to the final PCR results was estimated to be around 6 h. This estimation incorporated the time from sample collection to delivery to the lab (15 min), followed by sample processing to automatic DNA extraction (4 h), the execution of EG-mPCR (1.5 h), and concluding with data analysis (15 min).

## Results

### Detection of six bacteria and AMR genes by EG-mPCR assays

**Fig. 1:**
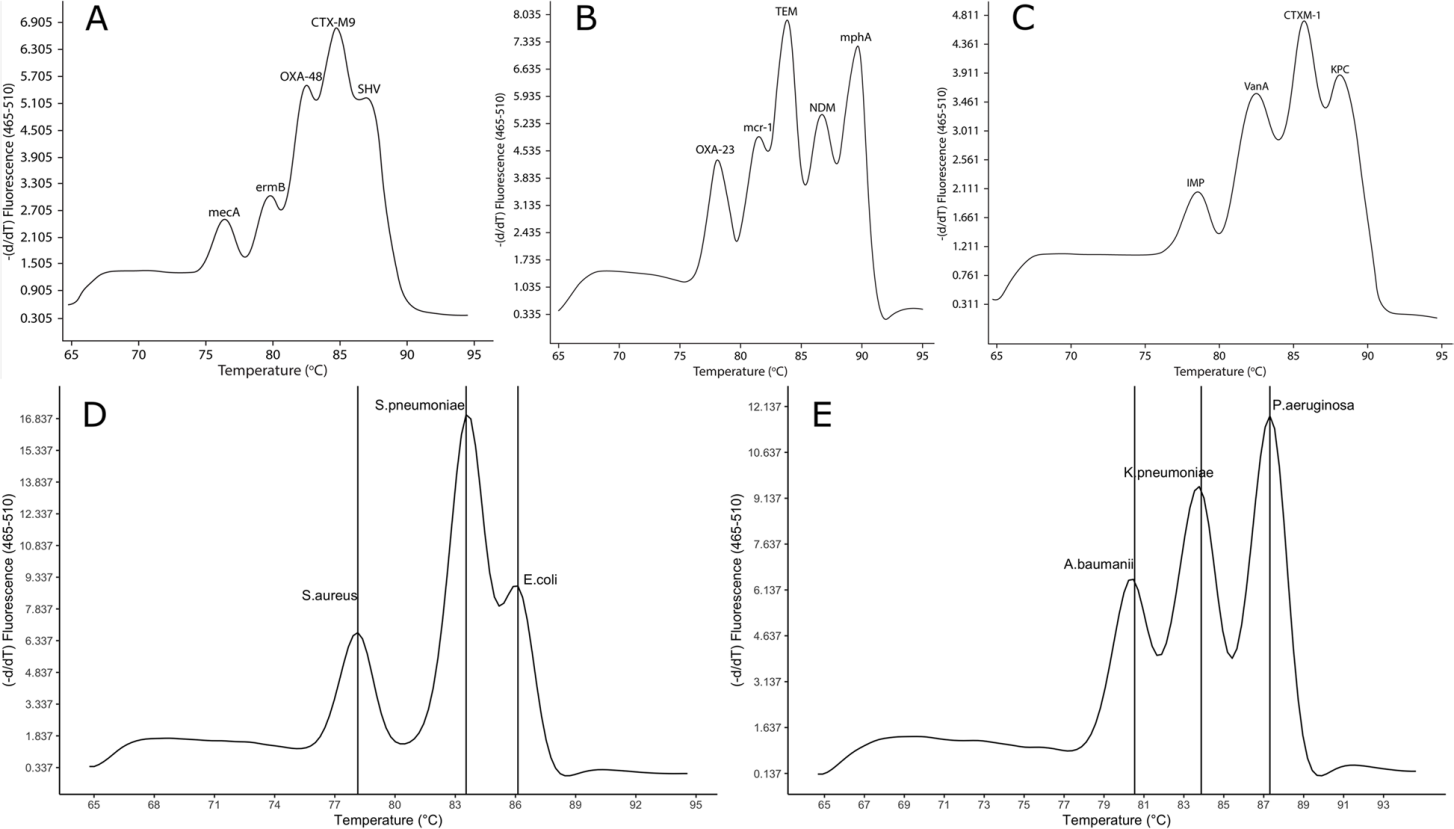
Melting curve analysis showing the melting temperature peaks (Tm) of 14 AMR genes (**A**, **B**, **C**) and 6 bacterial pathogens (**D**, **E**) and negative control (NTC) in 5 multiplex PCR assays

Two EG-mPCR assays were used to detect 6 bacterial pathogens and additional three EG-mPCR assays were used to detect 14 AMR genes (Table 1). In each EG-mPCR assay, the melting curve analysis showed distinct Tm peaks corresponding to the target bacteria and AMR genes, while negative controls did not show any signal (Fig. 1). The difference between two consecutive peaks was more than 2 °C (Table 2). There was no interference and cross-reactivity in our EG-mPCR assays. We additionally evaluated our assays on other closely related bacteria and did not find any non-specific signal for *Salmonella* Typhi*, Campylobacter jejuni, Acinetobacter lwoffii, Staphylococcus epidermidis*. However, the *yaiO* primer which was supposed to be specific to *E. coli*, produced false positive signal for one *Shigella sonnei* isolate.

**Table 2:** Tm values of primers used for singleplex and multiplex real-time PCR assays

Primer	Gene	Intra-assay Tm	Inter-assay Tm	Intra-assay CV%	Inter-assay CV%
**Pathogen detection assays**					
A. baumannii-F	ompA	80.6 ± 0.2	80.75 ± 0.27	0.3	0.3
A. baumannii-R					
P. aeruginosa-F	oatA	87.6 ± 0.3	87.7 ± 0.3	0.4	0.5
P. aeruginosa-R					
K. pneumoniae-F	khe	84.2 ± 0.3	84.22 ± 0.4	0.3	0.3
K. pneumoniae-R					
E. coli-F	yaiO	86.8 ± 0.1	86.95 ± 0.4	0.2	0.4
E. coli-R					
S. aureus-F	sa442	79 ± 0.16	79.12 ± 0.3	0.2	0.3
S. aureus-R					
S. pneumoniae-F	ply	84.49 ± 0.13	84.66 ± 0.3	0.1	0.4
S. pneumoniae-R					
**AMR gene detection assays**					
mecA-F	mecA	76.49 ± 0.14	76.44 ± 0.7	0.19	0.92
mecA-R					
ermB-F	ermB	79.83 ± 0.19	79.77 ± 0.73	0.24	0.91
ermB-R					
OXA-48-F	bla_OXA-48_	82.9 ± 0.08	81.98 ± 0.72	0.09	0.88
OXA-48-R					
CTX-M-9-F	bla_CTX-M-9_	84.72 ± 0.1	84.66 ± 0.69	0.12	0.81
CTX-M-9-R					
SHV-F	bla_SHV_	88.27 ± 0.1	87.85 ± 0.49	0.11	0.55
SHV-R					
OXA-23-F	bla_OXA-23_	78.26 ± 0.11	77.98 ± 0.39	0.14	0.51
OXA-23-R					
mcr-1-F	mcr-1	81.8 ± 0.13	81.43 ± 0.55	0.16	0.67
mcr-1-R					
TEM-F	bla_TEM_	83.77 ± 0.16	83.41 ± 0.55	0.19	0.66
TEM-R					
NDM-F	bla_NDM_	87.22 ± 0.11	86.87 ± 0.54	0.12	0.62
NDM-R					
mphA-F	mphA	89.43 ± 0.19	89.12 ± 0.46	0.22	0.51
mphA-R					
IMP-F	bla_IMP_	78.14 ± 0.15	78.68 ± 0.55	0.2	0.7
IMP-R					
VanA-F	VanA	82.21 ± 0.22	82.61 ± 0.42	0.26	0.51
VanA-R					
CTX-M-1-F	bla_CTX-M-1_	85.69 ± 0.04	86.15 ± 0.57	0.04	0.66
CTX-M-1-R					
KPC-F	bla_KPC_	88.78 ± 0.5	89.4 ± 0.63	0.57	0.7
KPC-R					

### Reproducibility, quantification and LOD of the two EG-mPCR assays for bacterial identification

The reproducibility of EG-mPCR assays was evaluated by accessing *T*_*m*_ values in inter and intra-assays. The standard deviation (SD) and coefficient variation (CV) of *T*_*m*_ were calculated in 5 replicates within the same run (intra-assay) and across 5 different runs (inter-assay). The intra-assay CV of each of the target bacteria ranged from 0.1% to 0.4% and the inter-assay CV ranged from 0.3% to 0.5%. Similarly, intra-assay CV and inter-assay CV of each of the target AMR genes varied from 0.04% to 0.57% and from 0.51% to 0.92%, respectively (Table 2). These data indicated good reproducibility of Tm values in our assays.

The standard curve for each bacterium in the two EG-mPCR assays was obtained by plotting the fluorescence intensity (− d*F*/d*T*) or the height of the peak (*y*-axis) values against the log_10_ of the bacterial concentrations (*x*-axis) inferred from the serially diluted DNA control mixes. The coefficient of determination of linear regression model of standard curves were *R*^2^ = 0.92 for *S. aureus*, *R*^2^ = 0.92 for *S. pneumoniae*, *R*^2^ = 0.91 for *E. coli*, *R*^2^ = 0.97 for *A. baumannii*, *R*^2^ = 0.98 for *K. pneumoniae* and *R*^2^ = 0.95 for *P. aeruginosa.* These data showed a good linear correlation between the fluorescence values and the log_10_ of bacterial concentration over a range of bacterial concentrations. The efficiency of EG-mPCR assay was 104% (95% CI, 88.6–119%) for the detection of *A. baumannii*/*P. aeruginosa*/*K. pneumoniae* and 92% (95% CI, 80–104%) for the detection of *E. coli/S. aureus/S. pneumoniae.*

**Fig. 2:**
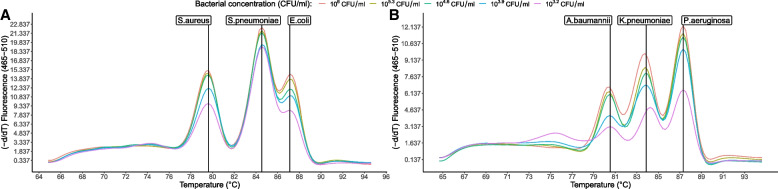
The sensitivity of simultaneous detection of *S. aureus*, *S. pneumoniae*, *E. coli* (**A**) and *A. baumannii*, *K. pneumoniae*, *P. aeruginosa* (**B**) with concentrations ranging 10^6^ to 12.8 CFU/mL. LOD of simultaneous detection of *S. aureus*, *S. pneumoniae*, *E. coli* was 1600 CFU/mL and the same for *A. baumannii, K. pneumoniae, P. aeruginosa*

The limit of detection (LOD) was evaluated by observing the Tm peaks of each bacterium in the two serially diluted DNA control mixes. The experiment was performed in 5 replicates within the same run and in 10 replicates between different runs. Based on the lowest concentration of a target bacterium in a control mix where all targets showed positive signal, a LOD of 1600 CFU/mL was determined for each of the two EG-mPCR assays (Fig. 2). When a TA specimen was spiked with target bacteria, a LOD of 3200 CFU/mL was identified for each of the two EG-mPCR assays.

### Diagnostic performance of EG-mPCR assays in comparison to conventional culture

Between December 2020 and March 2021, 50 consecutive TA and sputum specimens were included for both conventional culture and EG-mPCR assays. Conventional culture showed that 38 samples (76%) were positive for a single pathogen and 8 samples (16%) were positive for more than one pathogen (including 4 pathogens not covered in the PCR assays: *Haemophilus influenzae*, *Moraxella spp.*, *Stenotrophomonas maltophilia*, *Enterobacter. spp*). *P. aeruginosa* was the most prevalent pathogen (26%, 13/50), followed by *A. baumannii* (20%, 10/50), *S. aureus* (18%, 9/50), *K. pneumoniae* (12%, 6/50) and *E. coli* (2%, 1/50) (Table 3). Three samples (6%) were culture negative for all six pathogens. A total of 51 target pathogens were yielded from culture.

**Table 3:** Performance of EG-mPCR assays compared with the conventional culture method

Bacterial pathogens	EG-mPCR	Conventional culture method		% Sensitivity (95% CI)	% Specificity (95% CI)	% PPV (95% CI)	% NPV (95% CI)	Kappa
		Positive	Negative					
K. pneumoniae	positive	10	1	100 (69.2, 100)	97.5 (86.8, 99.9)	90.9 (58.7, 99.8)	100 (91.0, 100)	0.94
	negative	0	39					
A. baumannii	positive	14	1	100 (76.8, 100)	97.2 (85.5, 99.9)	93.3 (68.1, 99.8)	100 (90.0, 100)	0.95
	negative	0	35					
P. aeruginosa	positive	18	4	100 (81.5, 100)	87.5 (71.0, 96.5)	81.8 (59.7, 94.8)	100 (87.7, 100)	0.8
	negative	0	28					
E. coli	positive	2	2	100 (15.8, 100)	95.8 (85.7, 99.5)	50 (6.8, 93.2)	100 (92.3, 100)	0.65
	negative	0	46					
S. aureus	positive	7	2	63.6 (30.8, 89.1)	94.9 (82.7, 99.4)	77.8 (40.0, 97.2)	90.2 (76.9, 97.3)	0.63
	negative	4	37					
S. pneumoniae	positive	0	6	NA	NA	NA	NA	NA
	negative	0	44					

**Fig. 3:**
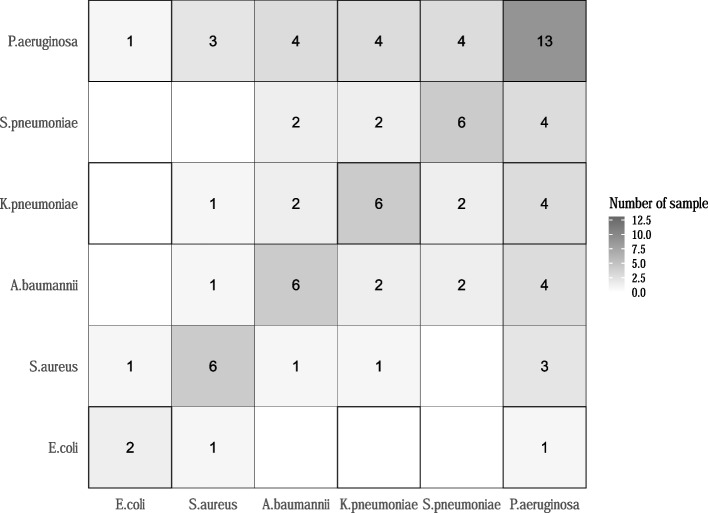
Heatmap of bacterial mixed infection pattern detected by the EG-mPCR assays in the respiratory samples

Compared to culture results, PCR assays found less samples positive for a single target pathogen (28/50, 56%) and more samples positive for at least two target pathogens (18/50, 36%). The most common patterns of co-infections by PCR were *P. aeruginosa* and *S. pneumoniae* (3/18) and *P. aeruginosa* and *S. aureus* (3/18), *P. aeruginosa* and *K. pneumoniae* (2/18), and *P. aeruginosa* and *A. baumannii* (2/18). *P. aeruginosa* (12/18) was the most common organism found in co-infections (Fig. 3). EG-mPCR assays showed negative results in four samples, three of which were also culture negative. The sample that was culture positive but was negative by EG-mPCR contained *S. aureus*. Overall, PCR assays detected more target bacteria than culture, with a total of 67 organisms.

The sensitivity and specificity of EG-mPCR assays versus culture were 100% and 97.5% for *K. pneumoniae*, 100% and 97.2% for *A. baumannii*, 100% and 87.5% for *P. aeruginosa*, 100% and 93.8% for *E. coli* and 63.6% and 94.9% for *S. aureus*, respectively (Table 3). The positive predictive value (PPV) and negative predictive value (NPV) of EG-mPCR assays were 90.9% and 100% for *K. pneumoniae*, 93.3% and 100% for *A. baumannii*, 81.8% and 100% for *P. aeruginosa*, 50% and 100% for *E. coli* and 77.8% and 90.2% for *S. aureus*, respectively.

**Fig. 4:**
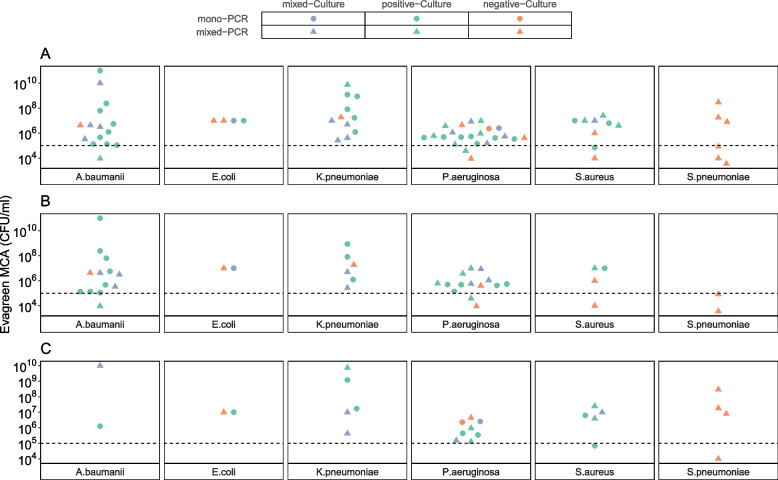
The detection and quantity of 6 target bacteria by EG-mPCR assays in accordance with culture results. **A** All samples; **B** Tracheal aspirates; **C** Sputum samples. The circles indicate the detection of the target bacterium alone, while the triangles illustrate co-detection with another target bacterium in EG-mPCR assays. Colours (green, purple, and orange) correspond to the culture results of the target bacterium: green for culture positive alone, purple for culture positive with another bacterium, and orange for culture negative. The Y-axis represents the target bacterium quantity in CFU/ml, determined through the quantitative EG-mPCR. The dashed line indicates the cut-off for culture positivity (10.^5^ CFU/ml)

Out of the 51 target pathogens detected by culture, 48 pathogens (94.1%) were also identified by EG-mPCR assays with a quantity ≥ 10^5^ CFU/mL, which is the culture positivity cut-off (Fig. 4A). These findings demonstrated a high degree of agreement between EG-mPCR assays and culture method. When considering only target pathogens detected by quantitative culture with growth ≥ 10^5^ CFU/mL in TA samples (*n* = 26), the concordant rate with EG-mPCR assays reached 100% (Fig. 4B)*.* There were some discordant results between the two methods. The PCR assays found 6 *S. pneumoniae* positives with a quantity ranging from 10^3.6^ to 10^9^ CFU/mL while culture method did not grow any *S. pneumoniae* isolates. Furthermore, the PCR assays identified an additional 10 target bacteria (8 bacteria with a quantity ≥ 10^5^ CFU/mL and 2 bacteria with a quantity < 10^5^ CFU/mL) that were missing by culture (Fig. 4A).

### Application of EG-mPCR assays for AMR gene detection from clinical samples

Three EG-mPCR assays were used to identify 14 AMR genes commonly present in the target pathogens from the 50 respiratory samples. After excluding mixed infections by PCR, the level of correlation between the presence of AMR genes and AMR phenotype was assessed in 28 target bacteria (Table 4, Figure S1). In *A. baumannii,* all seven isolates carrying *bla*_OXA23_ and *bla*_TEM_ were resistant to ceftazidime, cefepime and carbapenems (imipenem, meropenem). All four *K. pneumoniae* isolates that were resistant to 3rd and 4th- generation cephalosporins (ceftriaxone, cefotaxime, cefepime) carried *bla*_CTX-M-1_, three of them also carried *bla*_TEM_. *Bla*_NDM_ and *bla*_OXA48_ genes were both detected in two carbapenem resistant *K. pneumoniae* isolates. For *P. aeruginosa*, *bla*_OXA23_ was found in one isolate that was resistant to imipenem and meropenem and *bla*_CTX-M-9_ was found in another isolate that was resistant to the 3rd generation cephalosporin (ceftriaxone, cefotaxime). Among five *S. aureus* isolates that were resistant to macrolides (clindamycin), *ermB* gene was detected in 3 isolates (60%). Furthermore, *mecA* gene was found in 3/4 oxacillin-resistant *S. aureus*. Two isolates (1 *K. pneumoniae* and 1 *A. baumannii*) were resistant to colistin, but the *mcr-1* gene was not found. There was one *E. coli* isolate resistant to cefotaxime and imipenem carrying *bla*_SHV_ and *bla*_TEM_ genes but the carbapenem resistance genes were undetected.

**Table 4:** Frequency of common AMR genes detected in microbiologically-confirmed target bacteria either phenotypically resistant to 3rd/4th cephalosporins, carbapenem, macrolide and oxacillin

		**Cephalosporin resistance**		**Carbapenem resistance**		**Macrolide resistance**		**Oxacillin resistance**	
	Pathogens	Resistance	AMR genes	Resistance	AMR genes	Resistance	AMR genes	Resistance	AMR genes
	(n)	(n)	(n)	(n)	(n)	(n)	(n)	(n)	(n)
**Gram negative**	*A. baumannii* (9)	*7*	TEM (7/7)	*7*	OXA-23 (7/7)				
	*K. pneumoniae* (5)	*4*	TEM (3/4)	*2*	NDM (2/2)				
			CTXM-1 (1/4)		OXA-48 (2/2)				
	*E. coli* (2)	1	SHV (1/1)	1					
			TEM (1/1)						
	*P. aeruginosa* (9)	1	CTXM-9 (1/1)	1	OXA-23 (1/1)				
**Gram positive**	*S. aureus (5)					5	ermB (3/5)	4	mecA (3/4)

^*^EG-mPCR detected 3 *S. aureus* and did not identify 2 *S. aureus* in single-infection cases

## Discussion

The selection and duration of empirical antibiotic treatment for LRTIs have become a major challenge in LMIC settings where MDR causative bacteria are prevalent. Although culture-guided definitive treatment is often followed to avoid overuse/misuse of initial antibiotics, the long waiting time for culture results has delayed effective antibiotic therapy, leading to increased mortality and selection of AMR bacteria. Rapid and inexpensive molecular PCR-based diagnostics are urgently needed in clinical practice to inform antibiotic therapy. Here, we successfully developed two EG-mPCR assays to detect and quantify 6 major bacterial pathogens and three EG-mPCR assays to identify 14 AMR genes directly from TA and sputum samples. Our PCR assays exhibited high sensitivity (100%) and high specificity (from 87.5% to 97.5%) compared to conventional culture, except for *S. aureus* (sensitivity: 63.6%). The sensitivity, specificity, and agreement rates in this study are comparable to the results of other molecular assays for the diagnostics of respiratory agents [14]. The FDA-cleared CE-marked BioFire FilmArray Pneumonia Panel had 100% sensitivity for 15/22 etiologic targets with BAL specimens and 10/24 targets with sputum specimens. Sensitivities for other targets were either 75% or could not be calculated due to their low prevalence in the study population. The specificity for all targets was 87.2% [14]. Despite its high diagnostic yield, the direct cost of FilmArray Pneumonia Panel was estimated to be around €155/test [17] and its clinical utility in LMIC required further evaluation [15]. Our assays are simple and inexpensive with a turnaround time of around 6 h, offering a useful tool to potentially guide optimal antibiotic therapy.

A key advantage of our PCR assays is the ability to quantify the amount of each bacterium and thus may help to distinguish between bacterial colonization and infection. In fact, the quantitative data obtained from PCR assays demonstrated 100% concordance with the quantitative culture results of TA samples. A previous study using a similar approach raised concern about the reduced linear relationship between fluorescence levels and bacterial concentration in multiplex PCR assays, which may be attributed to the competition amongst EvaGreen dyes [25]. This limitation has been addressed in this work by increasing the amount of the PCR mix and adding extra EvaGreen dye to the reaction. Our experiments showed a good linear correlation (*R*^2^ > 0.91) between fluorescence readouts and the log10 of bacterial concentrations. Furthermore, the LOD of PCR assays (10^3.6^ CFU/mL) was much lower than the cut-off point for culture positivity (≥ 10^5^ CFU/mL), meaning our assays not only could identify true pathogens above the positivity cut-off but also detect other potentially pathogenic bacteria present with lower concentrations for further monitoring.

Notably, PCR assays were able to detect more mixed target bacteria in the respiratory samples. The fact that EG-mPCR assays were more sensitive than conventional culture in detecting mixed infections of which most target bacteria had a concentration > 10^5^ CFU/mL suggested that culture method may have missed some true target pathogens in the sample. Although culture is considered to be the gold standard for the diagnosis of bacterial LRTIs, it may be challenging to accurately recover all pathogens from a nonsterile sample [26]. Alternatively, recent antibiotic treatment may affect the culture results and some target bacteria may present at a concentration < 10^5^ CFU/mL and thus not detected by the culture method [14, 17, 18]. Mixed bacteria by PCRs with quantitative results above the culture positivity cut-off can be regarded as a recent or current mixed infection. On the other hand, mixed bacteria by PCRs with the quantitative result(s) below the cut-off should be followed up by additional microbiological culture and PCR testing to confirm the pathogens. We recommend promptly reporting the PCR results to clinicians for patient monitoring and advocating for further testing if deemed necessary.

In this study, *S. pneumoniae* was only found by PCR assays in mixed infections, which was the main factor affecting the agreement between the two methods. *S. pneumoniae* was notoriously difficult to identify by conventional culture because the bacteria tends to autolyze after reaching the stationary phase, as well as the effect of prior antibiotic treatment [27]. Previous studies comparing multiplex PCR with culture for the detection of *S. pneumoniae* and *H. influenzae* from sputum also found similar results with *S. pneumoniae* only found by qPCR [28]. Our quantitative PCR assay therefore could serve as a useful diagnostic tool to detect *S. pneumoniae* from respiratory samples.

The use of EG-mPCR assays for AMR gene identification was also evaluated using 50 clinical samples. Overall, our findings demonstrated a high agreement between the detected AMR genes and the AMR profiles of target bacteria present in the single infections. This means our assays can rapidly identify clinically relevant AMR genes to guide antibiotic therapy. As PCRs tend to detect more mixed infections, there may be some uncertainty about which AMR genes belong to which bacteria in these samples. However, further research with increased sample size can add more insights into the species-specific AMR gene distribution in single infections, and thus help to predict the most probable combinations of bacterial species-AMR genes in mixed infections.

Our study has some limitations. The EG-mPCR assay had low sensitivity for the detection of *S. aureus,* probably due to its resistance to chemical lysis during DNA extraction [29]. Our assay should be further developed prior to its implementation for the diagnosis of *S. aureus*. Further optimization is currently underway, aiming to enhance the recovery of *S. aureus* DNA by incorporating lysozyme and lysostaphin during DNA extraction or omitting the sample processing step. Here, we only looked for commonly acquired AMR genes in the target bacteria, disregarding the resistance mechanisms driven by single point mutations or overexpression of efflux pump, and thus the absence of AMR genes may not always correlate with a lack of phenotype. Furthermore, as we only target 6 key pathogens, the PCR-negative samples may contain other pathogens that are not included in our PCR assays. Nevertheless, these bacteria are the main pathogens causing LRTIs in our setting. While our in-house EG-mPCR assays are more cost-effective than commercially available molecular testing systems, it is important to note that a comprehensive cost analysis was not conducted in this study.

## Conclusions

Our multiplex RT-PCR with EvaGreen dye MCA assays have been proven to be sensitive and quantitative for rapid detection of 6 key pathogens and 14 AMR genes directly from respiratory samples. Our assays provide culture-independent information regarding bacterial pathogens and pathogen abundance in samples as well as the genotypic AMR status of LRTIs. Our PCR method can possibly offer a tool to promote antibiotic stewardship and evaluate antibiotic treatment response in patients with LRTIs. The PCR assays have strong potentials to be adopted in clinical practice due to its feasibility, low cost and fast turnaround time. The impact of our PCR assays on antibiotic therapy and clinical outcome warrants a thorough investigation to facilitate its implementation in routine practice.

### Supplementary Information

**Additional file 1:** **Table S1. **Primer sequences used for conventional multiplex PCR assays [30–38].

**Additional file 2: Figure S1.** The antimicrobial susceptibility testing results of target bacteria.

## Data Availability

Raw dataset of the present study are available from the corresponding author on reasonable request.

## Abbreviations

AMR: Antimicrobial resistance
LRTIs: Lower Respiratory Tract Infections
PCR: Polymerase Chain Reaction
RT-PCR: Real-time Polymerase Chain Reaction
LOD: Limit of Detection
ICU: Intensive Care Unit
MDR: Multidrug resistance
LMIC: Low- and middle-income country
MCA: Melting Curve Analysis
EG: EvaGreen
ATCC: American Type Culture Collection
EG-mPCR: Evagreen-based multiplex real-time PCR with MCA
LB: Lubria-Bertani
NA: Nutrient Agar
TA: Tracheal Aspirate
OUCRU: Oxford University Clinical Research Unit
MRD: Maximum Recovery Diluent
EG-mPCR: Evagreen-based
CV: Coefficient variation
SD: Standard deviation
PPV: Positive predictive value
NPV: Negative predictive value
CFU: Colony Forming Unit
