# Supplementary material for: Development and validation of multiplex real-time PCR for simultaneous detection of six bacterial pathogens causing lower respiratory tract infections and antimicrobial resistance genes
Source: BMC Infect Dis. 2024 Feb 7;24:164. doi: 10.1186/s12879-024-09028-2 (PMC10848345; doi:10.1186/s12879-024-09028-2)
Supplement: Supplementary file 1 — Additional file 1: Table S1. Primer sequences used for conventional multiplex PCR assays [30–38]. [file 12879_2024_9028_MOESM1_ESM.docx]

**Table S1.** Primer sequences used for conventional multiplex PCR assays

| **Primer** | **Gene** | **Sequence (5'-3')** | **Final conc.(μM)** | **Amplicon size (bp)** | **Reference** |
| --- | --- | --- | --- | --- | --- |
| **Pathogen detection**  **assays** | | | | | |
| A. baumannii-F | bla_OXA-51-like_ | AGTGAAGCGTGTTGGTTAT | 0.5 | 285 | (31) |
| A. baumannii-R |  | CAGCCTACTTGTGGGTYTA |  |  |  |
| P. aeruginosa-F | oatA | CTGGGTCGAAAGGTGGTTGTTATC | 0.5 | 232 | (32) |
| P. aeruginosa-R |  | GCGGCTGGTGCGGCTGAGTC |  |  |  |
| K. pneumoniae-F | khe | GATCCCGCAGAACATGAG | 0.5 | 755 | this study |
| K. pneumoniae-R |  | GTTCTTCCCGGTTGGTGATA |  |  |  |
| E. coli-F | yaiO | TGATTTCCGTGCGTCTGAATG | 0.5 | 115 | (20) |
| E. coli-R |  | ATGCTGCCGTAGCGTGTTTC |  |  |  |
| S. aureus-F | sa442 | TCGGTACACGATATTCTTCAC | 0.5 | 179 | (18) |
| S. aureus-R |  | ACTCTCGTATGACCAGCTTC |  |  |  |
| S. pneumoniae-F | ply | ATTTCTGTAACAGCTACCAACGA | 0.5 | 348 | (18) |
| S. pneumoniae-R |  | GAATTCCCTGTCTTTTCAAAGTC |  |  |  |
| **AMR gene detection**  **assays** | | | | | |
| mecA-F | mecA | GGCATCGTTCCAAAGAATGT | 0.5 | 551 | this study |
| mecA-R |  | AGTGGAACGAAGGTATCATCTT |  |  |  |
| ermB-F | ermB | GAAAAGGTACTCAACCAAATA | 0.5 | 639 | (33) |
| ermB-R |  | AGTAACGGTACTTAAATTGTTTAC |  |  |  |
| OXA-48-F | bla_OXA-48_ | TTGGTGGCATCGATTATCGG | 0.5 | 743 | (34) |
| OXA-48-R |  | GAGCACTTCTTTTGTGATGGC |  |  |  |
| CTX-M-9-F | bla_CTX-M-9_ | CAAAGAGAGTGCAACGGATG | 0.5 | 205 | (35) |
| CTX-M-9-R |  | ATTGGAAAGCGTTCATCACC |  |  |  |
| SHV-F | bla_SHV_ | AGCCGCTTGAGCAAATTAAAC | 0.5 | 713 | (36) |
| SHV-R |  | ATCCCGCAGATAAATCACCAC |  |  |  |
| OXA-23-F | bla_OXA-23_ | GATCGGATTGGAGAACCAGA | 0.5 | 501 | (37) |
| OXA-23-R |  | ATTTCTGACCGCATTTCCAT |  |  |  |
| mcr-1-F | mcr-1 | AGTCCGTTTGTTCTTGTGGC | 0.5 | 320 | (38) |
| mcr-1-R |  | AGATCCTTGGTCTCGGCTTG |  |  |  |
| TEM-F | bla_TEM_ | GAGTATTCAACATTTCCGTGTC | 0.5 | 850 | (34) |
| TEM-R |  | TAATCAGTGAGGCACCTATCTC |  |  |  |
| NDM-F | bla_NDM_ | GGTTTGGCGATCTGGTTTTC | 0.5 | 621 | (37) |
| NDM-R |  | CGGAATGGCTCATCACGATC |  |  |  |
| mphA-F | mphA | AACTGTACGCACTTGC | 0.5 | 837 | (33) |
| mphA-R |  | GGTACTCTTCGTTACC |  |  |  |
| IMP-F | bla_IMP_ | GGAATAGAGTGGCTTAAYTCTC | 0.5 | 232 | (39) |
| IMP-R |  | GGTTTAAYAAAACAACCACC |  |  |  |
| CTX-M-1-F | bla_CTX-M-1_ | AAAAATCACTGCGCCAGTTC | 0.5 | 421 | (35) |
| CTX-M-1-R |  | AGCTTATTCATCGCCACGTT |  |  |  |
| KPC-F | bla_KPC_ | ATCTGACAACAGGCATGACG | 0.5 | 155 | (40) |
| KPC-R |  | GTCCAGACGGAACGTGGTAT |  |  |  |
