# Supplementary material for: Development and validation of multiplex real-time PCR for simultaneous detection of six bacterial pathogens causing lower respiratory tract infections and antimicrobial resistance genes
Source: BMC Infect Dis. 2024 Feb 7;24:164. doi: 10.1186/s12879-024-09028-2 (PMC10848345; doi:10.1186/s12879-024-09028-2)
Supplement: Supplementary file 2 [file 12879_2024_9028_MOESM2_ESM.docx]

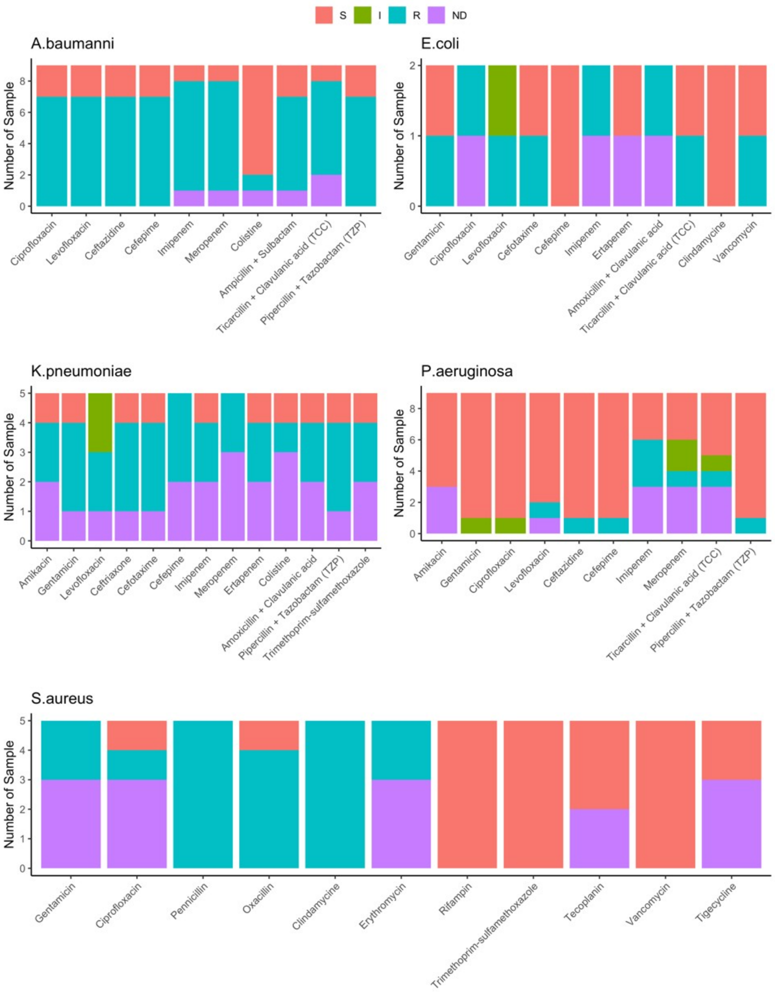

Figure S1. The antimicrobial susceptibility testing results of target bacteria
